# Transcriptomic Profiling Associates Collagen‐Rich Extracellular Matrix With Postoperative Angiogenesis in Moyamoya Disease

**DOI:** 10.1111/jcmm.71267

**Published:** 2026-08-27

**Authors:** Jing‐yi Chen, Shun‐fei Xu, Zi‐qing Wang, Wen‐xin Jin, Ri‐sheng Liang, Xian‐kun Tu

**Affiliations:** ^1^ Department of Neurosurgery Fujian Medical University Union Hospital Fuzhou Fujian China

**Keywords:** angiogenesis, collagen, extracellular matrix, moyamoya disease, temporal muscle

## Abstract

Indirect revascularization is an effective treatment for moyamoya disease (MMD), but the surgery effect depends on the collateral vessel formation. This study aimed to explore the molecular mechanisms associated with angiogenesis in the temporal muscle post‐surgery. We employed a single‐centre, prospective sampling, retrospective cohort design. Temporal muscle tissues were harvested during surgery, and comprehensive transcriptome‐wide RNA sequencing was performed. Patients were categorized into Good (Matsushima Grade A, *n* = 13) and Poor (Matsushima Grade C, *n* = 12) collateralization groups based on postoperative angiography. Differentially expressed genes (DEGs) were analysed, and key findings were validated via quantitative real‐time PCR (qPCR). A total of 25 patients were enrolled. Transcriptomic profiling revealed that pathways related to the extracellular matrix (ECM) and collagen family were significantly enriched in the Good group, suggesting a pivotal role for collagens in promoting postoperative angiogenesis. Subgroup analysis revealed distinct transcriptional profiles: paediatric patients exhibited a cluster characterized by high collagen expression alongside altered expression of genes related to mitochondrial electron transport. qPCR validation confirmed the upregulation of the core ECM component COL1A1 and the downregulation of the matrix‐degrading enzyme MMP3 in the Good group, indicating active ECM remodelling within the temporal muscle. Collectively, these findings suggest that a collagen‐rich ECM signature and the balance between collagen deposition and remodelling may modulate surgical efficacy in MMD.

## 1 Introduction

Moyamoya disease (MMD) is a cerebrovascular disorder characterized by progressive stenosis of the internal carotid artery terminus and the formation of a fragile, compensatory vessel network at the brain base. This stenosis reduces cerebral blood flow, causing ischemic events; the resulting hemodynamic stress can also lead to intracranial haemorrhage [1].

Recent transcriptomic studies have provided insights into the pathological mechanisms of MMD vasculopathy, highlighting key roles for extracellular matrix (ECM) remodelling, vascular smooth muscle cell phenotypic alteration, and immune‐vascular crosstalk [2, 3, 4, 5, 6]. For instance, recent single‐cell profiling of the superficial temporal artery reveals that immune cells, particularly natural killer T cells, promote vascular occlusion via macrophage migration inhibitory factor (MIF) signalling [2]. Moreover, emerging evidence indicates that aberrant activation of adhesion signalling pathways within the ECM microenvironment contributes significantly to the disease process [6].

Indirect revascularization, encompassing procedures such as encephaloduroarteriomyosynangiosis, is an effective treatment option for MMD. This surgical approach aims to reduce the risk of subsequent stroke and rebleeding. An encephaloduroarteriomyosynangiosis involves placing a temporal muscle graft onto the brain surface to stimulate spontaneous angiogenesis and facilitate collateral vessel formation. Unlike direct revascularization surgery, which can provide immediate augmentation of blood flow, indirect surgery takes more time to introduce angiogenesis from the temporal muscle [7]. Nevertheless, approximately half of MMD patients exhibit inadequate angiogenesis sprouting from the temporal muscle [8]. Consequently, the primary objective of this study is to identify the molecular correlates of postoperative angiogenesis within the temporal muscle graft.

Previous studies have shown that vascular endothelial growth factor, endothelial progenitor cells, Caveolin‐1, and other factors observed in patients with Moyamoya disease may play a key role in the generation of collateral vessels after indirect revascularization through endothelial hyperplasia and smooth muscle migration [9]. Clinical studies have identified several predictors of good collateral vessel formation, such as ischemic onset, abundant ICA moyamoya vessels, anterior haemorrhage, use of atorvastatin and aspirin, and childhood onset [8, 10, 11, 12, 13]. Studies have demonstrated that exogenous factors can enhance angiogenesis in this context. Date I et al. reported that vascular endothelial growth factor (VEGF) and apelin significantly promoted neovascularization [14, 15]. Jun‐ichi Kuratsu et al. reported that administration of granulocyte colony‐stimulating factor (G‐CSF) can enhance angiogenesis induced by indirect revascularization [16]. Chen et al. demonstrated that increasing miR‐126‐5p expression in the temporal muscle can promote angiogenesis [17].

Despite the efficacy of these external stimuli, the intrinsic molecular mechanisms that regulate angiogenesis within the temporal muscle after surgery remain poorly defined. Given that the temporal muscle serves as the donor tissue for indirect bypass, we hypothesized that its intrinsic transcriptomic profile may predict postoperative angiogenic success.

Therefore, we procured temporal muscle samples from 25 patients with MMD undergoing revascularization surgery. A transcriptomic analysis was conducted to gain insights into the underlying mechanisms of vascular growth and identify potential therapeutic targets.

To validate the transcriptomic findings and investigate the role of ECM remodelling in vascular growth, we conducted qPCR on selected differentially expressed genes. This panel included matrix metalloproteinases (MMP2, MMP3, MMP13, MMP14) and collagen family members (COL1A1, COL3A1, COL5A1, etc.), which are key regulators of ECM dynamics and vascular formation. This qPCR validation aimed to access the expression patterns between patients with good versus poor angiogenesis, thereby offering molecular insights into temporal muscle–derived revascularization.

## 2 Methods

### 2.1 Ethics Statement

This study was performed in accordance with the ethical guidelines of the 1975 Declaration of Helsinki and was approved by the Ethics Committee of Fujian Medical University Union Hospital. (2022YF008‐01). Written informed consent was obtained from all participants prior to their inclusion in the study.

### 2.2 Study Design and Patient Enrollment

**FIGURE 1:**
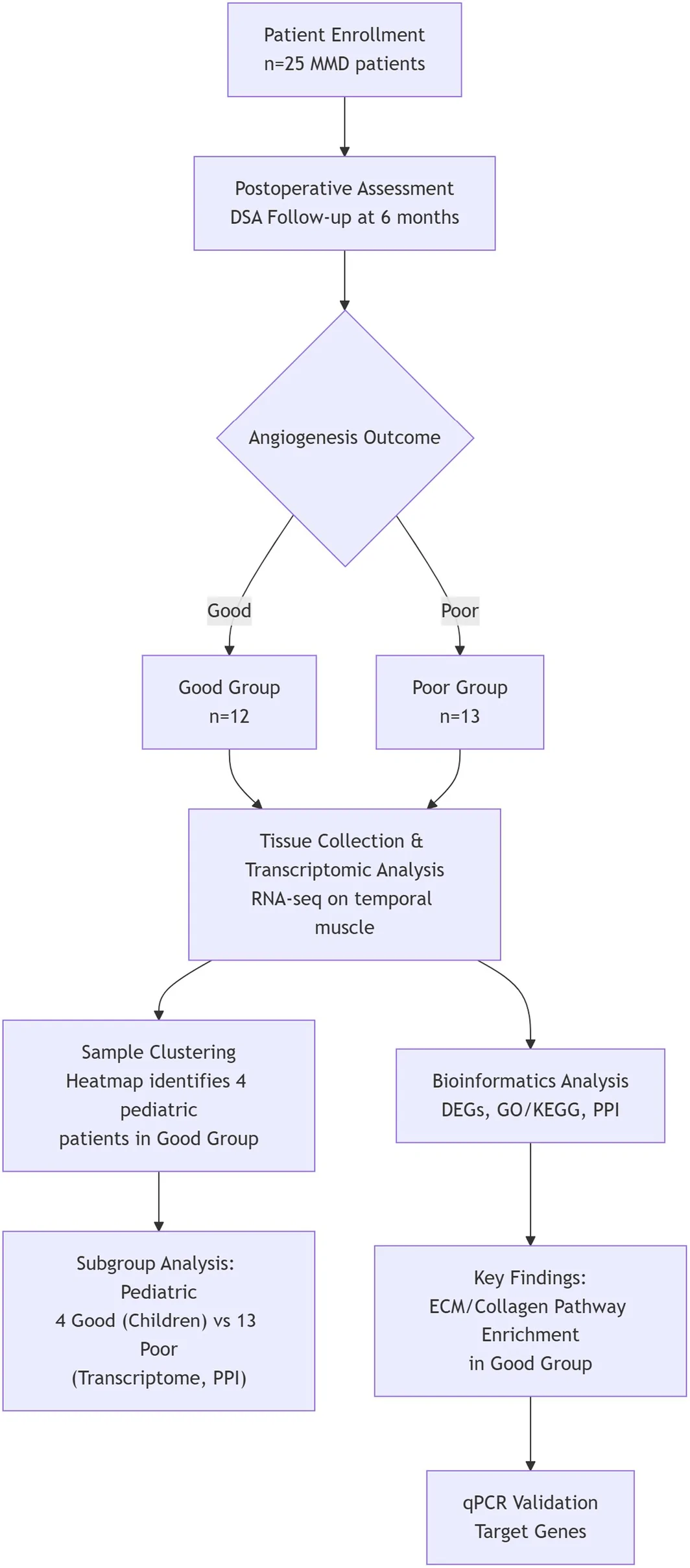
Study Design Flowchart.

This was a single‐centre, prospectively sampled, retrospective cohort study. Participants were recruited from Fujian Medical University Union Hospital from February 2022 to March 2023 (Figure 1). Patients diagnosed with MMD were included based on the 2012 Guidelines for the Diagnosis and Treatment of Moyamoya Disease. A total of 25 patients who underwent surgical treatment were enrolled. Patients with poor general conditions, recent cerebral ischemic stroke, or intracerebral haemorrhage were excluded. All patients received indirect revascularization surgery (encephaloduroarteriomyosynangiosis) and underwent DSA follow‐up at least 6 months postoperatively to assess collateral vessel formation.

### 2.3 Evaluation of Collateral Vessel Formation

Collateral formation was evaluated using the Matsushima grading system: Grade A, abundant collaterals covering > 2/3 of the MCA territory; Grade B, covering 1/3 to 2/3; and Grade C, covering 0 to 1/3. For analysis, patients with Grade A were assigned to the Good group, and Grade C to the Poor group. Patients with Grade B were excluded [18].

### 2.4 Collection of Temporal Muscle Samples

During surgery, the temporal muscle flap was carefully dissected. A section of the temporal muscle was excised and immediately rinsed with normal saline, then snap‐frozen in liquid nitrogen. Samples were stored at −80°C until further analysis.

### 2.5 RNA Sequencing and Bioinformatics Analysis

Total RNA concentration and purity were assessed using a NanoDrop 2000 (Thermo Fisher). RNA integrity was verified using an Agilent 2100 Bioanalyzer (RNA 6000 Nano Kit). Total RNA (≥ 1 μg) was used for library preparation with the NEBNext Ultra II RNA Library Prep Kit for Illumina. mRNA was enriched using Oligo (dT) magnetic beads and fragmented. First‐strand cDNA was synthesized using random hexamers, followed by second‐strand synthesis. The library was purified, end‐repaired, A‐tailed, and ligated with sequencing adapters. Fragments around 400–500 bp were selected using AMPure XP beads. The final library was amplified and validated using a High Sensitivity DNA Kit on the Agilent 2100 Bioanalyzer. Library quantification was performed using PicoGreen (Quantifluor‐ST) and QPCR (StepOnePlus Real‐Time PCR Systems).

Raw reads were filtered using Cutadapt (Q20 threshold). Clean reads were mapped to the human reference genome (GRCh38) using HISAT2 (v2.0.5). Gene counts were calculated using HTSeq (v0.9.1). Expression levels were normalized using FPKM. DEGs were identified using DESeq2 based on nominal *p‐*value < 0.05 and |log2FoldChange| > 1. Genes meeting these criteria were used as input for downstream functional enrichment analyses. Functional enrichment was performed using GO (topGO) and KEGG (clusterProfiler). Protein–protein interaction (PPI) networks were constructed using STRING (score ≥ 0.95) and visualized with Cytoscape (v3.9.1). Module analysis was conducted using the MCODE plugin.

### 2.6 Quantitative Real‐Time PCR

Total RNA was extracted from temporal muscle tissues using the RNA Extraction Kit (Servicebio, G3013). Tissue samples were homogenized using a three‐dimensional frozen grinder (Servicebio, KZ‐5F‐3D) with zirconium oxide grinding beads. RNA integrity and concentration were assessed using a NanoDrop 2000 spectrophotometer (Thermo Fisher Scientific). Genomic DNA contamination was removed, and complementary DNA (cDNA) was synthesized from 1 μg of total RNA using the SweScript All‐in‐One RT SuperMix for qPCR (Servicebio, G3337). The reverse transcription procedure was performed on an ETC811 PCR system (Dongsheng Innovation) as follows: 25°C for 5 min, 42°C for 30 min, and 85°C for 5 s. Quantitative real‐time PCR (qPCR) was performed using the 2X Universal Blue SYBR Green qPCR Master Mix (Servicebio, G3326) on a CFX Connect Real‐Time PCR Detection System (Bio‐Rad). The reaction system (15 μL) consisted of 7.5 μL Master Mix, 1.5 μL primers (2.5 μM), 2.0 μL cDNA template, and 4.0 μL Nuclease‐Free Water. The thermal cycling conditions were: 95°C for 30 s; followed by 40 cycles of 95°C for 15 s and 60°C for 30 s. Melting curve analysis was performed from 65°C to 95°C to verify product specificity. GAPDH was used as the internal reference gene. The relative expression levels of target genes were calculated using the 2^−ΔΔCt^ method.

### 2.7 Statistical Analysis

Categorical variables are presented as *n* (%) and compared using the Chi‐square test or Fisher's exact test. Continuous variables are expressed as mean ± standard deviation and compared using Student's *t*‐test or Mann–Whitney *U* test, as appropriate. Statistical significance was set at *p* < 0.05. Significance levels in figures are denoted as: *p* < 0.05, *p* < 0.01, *p* < 0.001. All statistical analyses were performed using SPSS version 27.0.1.

## 3 Results

### 3.1 Clinical Characteristics of Participants

A total of 25 patients were enrolled in this study, with 12 patients assigned to the Good group and 13 patients assigned to the Poor group (Table 1). Notably, the average age was significantly lower in the Good group.

**TABLE 1:** Baseline characteristics of patients in good and poor angiogenesis groups.

	All patients (*n* = 25)	Good group (*n* = 12)	Poor group (*n* = 13)	*p*
Female sex (no [%])	16 (64)	10 (83.33)	6 (46.15)	0.097^a^
Age, year	38.76 ± 18.10	29.25 ± 18.10	47.54 ± 9.70	0.007^b^
Type				
Ischemic (no [%])	16 (64)	9 (75)	7 (53.84)	0.411^a^
Haemorrhage (no [%])	9 (36)	3 (25)	6 (46.15)	
Medical history				
Diabetes (no [%])	4 (16)	1 (8.33)	3 (23.08)	0.593^a^
Hypertension (no [%])	8 (32)	3 (25.00)	5 (38.46)	0.673^a^
BMI (exclude 4 children)	24.43 ± 2.25 (*n* = 21)	24.33 ± 2.90 (*n* = 8)	24.49 ± 1.87 (*n* = 13)	0.897^b^

^a^ Fisher's exact test.

^b^ Student *t‐*test.

### 3.2 Transcriptomic Profiling of Temporal Muscle

**FIGURE 2:**
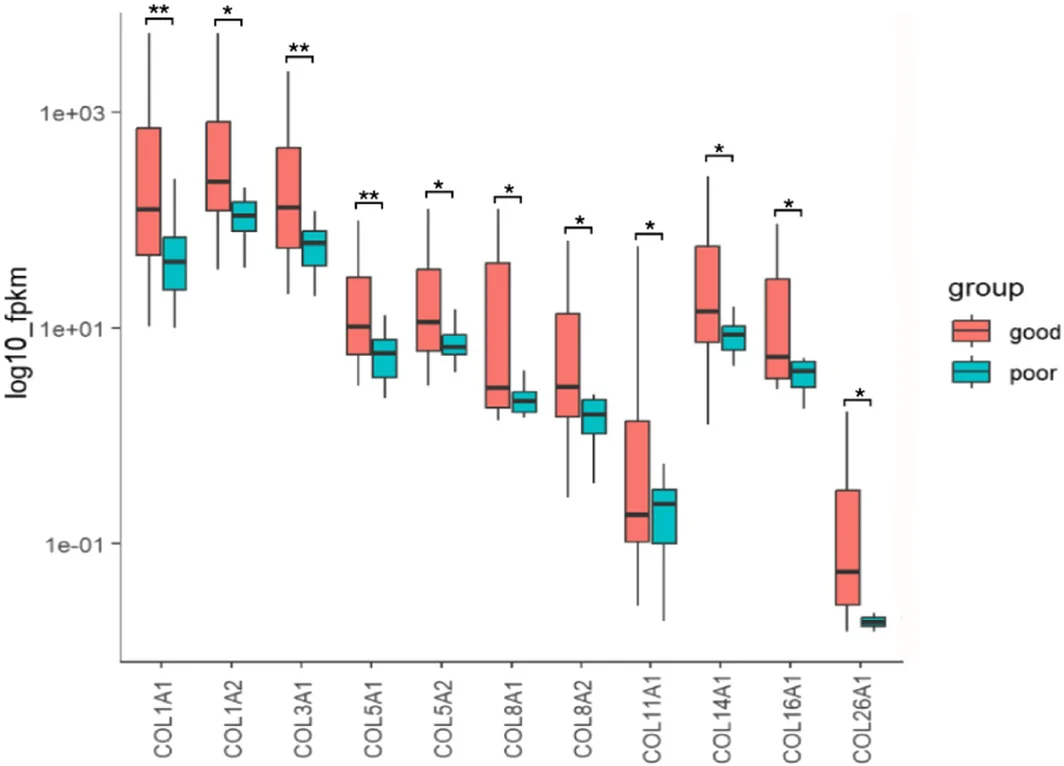
Boxplot representation of collagen family gene expression based on FPKM values.

We identified 530 DEGs (407 upregulated, 123 downregulated in the Good group). Subsequent network analysis of these DEGs using Cytoscape and the MCODE plugin identified three highly interconnected modules (Table 2). Notably, the top‐scoring module (score = 7.250) primarily consisted of core collagen genes, including COL1A1, COL1A2, COL3A1, COL5A1, and COL5A2, reinforcing the central role of the collagen family in the molecular signature of the Good group. This finding is consistent with the marked elevation of collagen family gene expression observed in the Good group (Figure 2), and transcriptomic analysis also revealed significant differential expression within the MMP family; specifically, MMP3 was notably downregulated in the Good group, suggesting distinct regulatory mechanisms for matrix remodelling.

**TABLE 2:** Details of top 3 modules from the PPI network.

Modules		Nodes/edges	Score	Genes
Top 3 PPI modules (derived from DEGs of good vs. poor groups, *n =* 25)				
1		9 nodes/29 edges	7.250	COL5A2, COL1A1, COL3A1, COL1A1, SERPINH1, PLOLCE, BMP1, COL5A1, ADAMTS2
2		4 nodes/5 edges	3.333	AGRN, SDC1, GPC3, BGN
3		3 nodes/3 edges	3.000	GPX8, SOD3, SOD2
Top 3 PPI modules (derived from DEGs of 4 children vs. poor groups, *n* = 17)				
1		17 nodes/135 edges	16.875	NDUFS2, NDUFA7, NDUFB2, NDUFB9, NDUFC1, NDUFAB1, NDUFB5, NDUFA1, NDUFS1, NDUFS3, NDUFB3, NDUFA9, NDUFB4, NDUFS7, NDUFB10, NDUFV2, NDUFA4
2		14 nodes 82 edges	12.615	TNNT1, TNNC2, TPM1, MYL2, TPM2, MYL1, TNNI2, MYL3, TTN, TNNI1, TPM3, ACTN2, TNNI3, MYH3
3		13 nodes 59 edges	9.833	COL6A1, COL4A1, COL1A1, COL5A1, COL6A3, P3H1, SERPINH1, COL4A2, COL5A2, COL12A1, COL6A2, COL3A1, COL1A2

**FIGURE 3:**
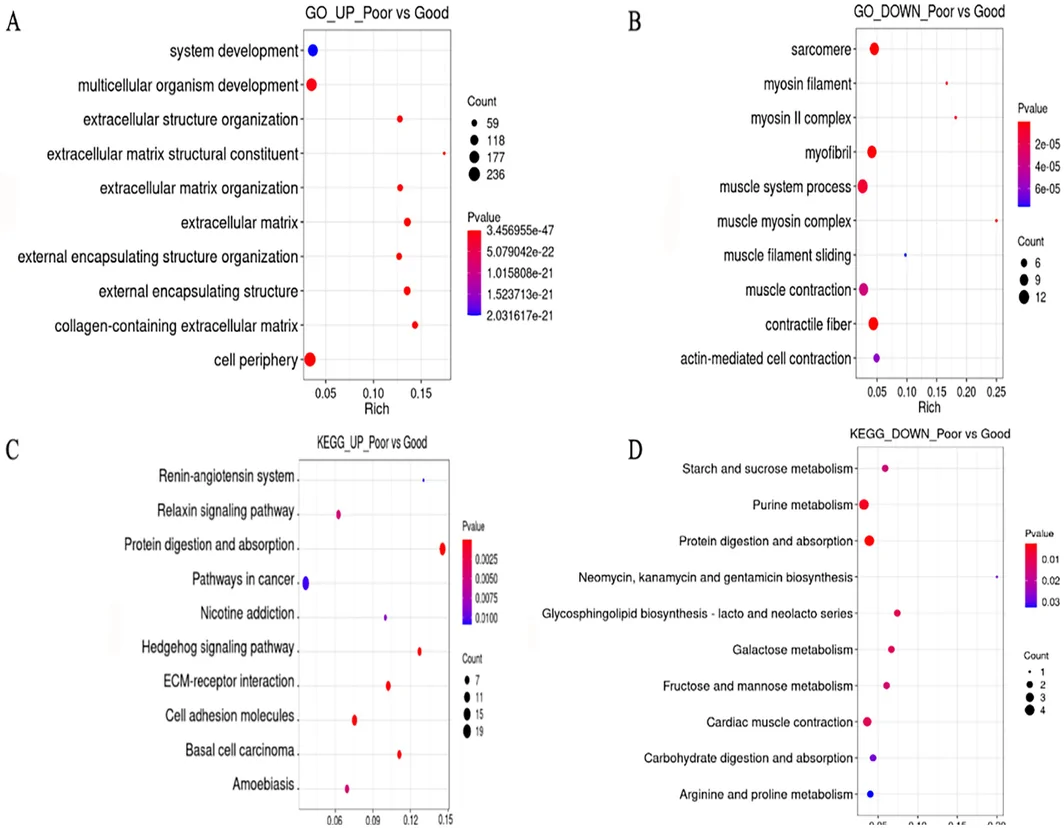
Comparison of good vs. poor group in GO and KEGG pathways. (A) Enrichment of upregulated genes in good group via GO analysis. (B) Enrichment of downregulated genes in good group via GO analysis. (C) KEGG pathway analysis of upregulated genes in the good group. (D) KEGG pathway analysis of downregulated genes in the good group.

Mechanistically, functional enrichment revealed that upregulated genes were enriched in ECM organization and collagen‐containing ECM (Figure 3A), contrasting with downregulated genes involved in muscle contraction and myofibril assembly (Figure 3B). Pathway analysis corroborated these findings, linking upregulated genes to ECM‐receptor interaction and cell adhesion (Figure 3C). Conversely, downregulated genes were associated with antibiotic biosynthesis and carbohydrate metabolism (Figure 3D).

### 3.3 Subgroup Analysis: Paediatric Patients

**FIGURE 4:**
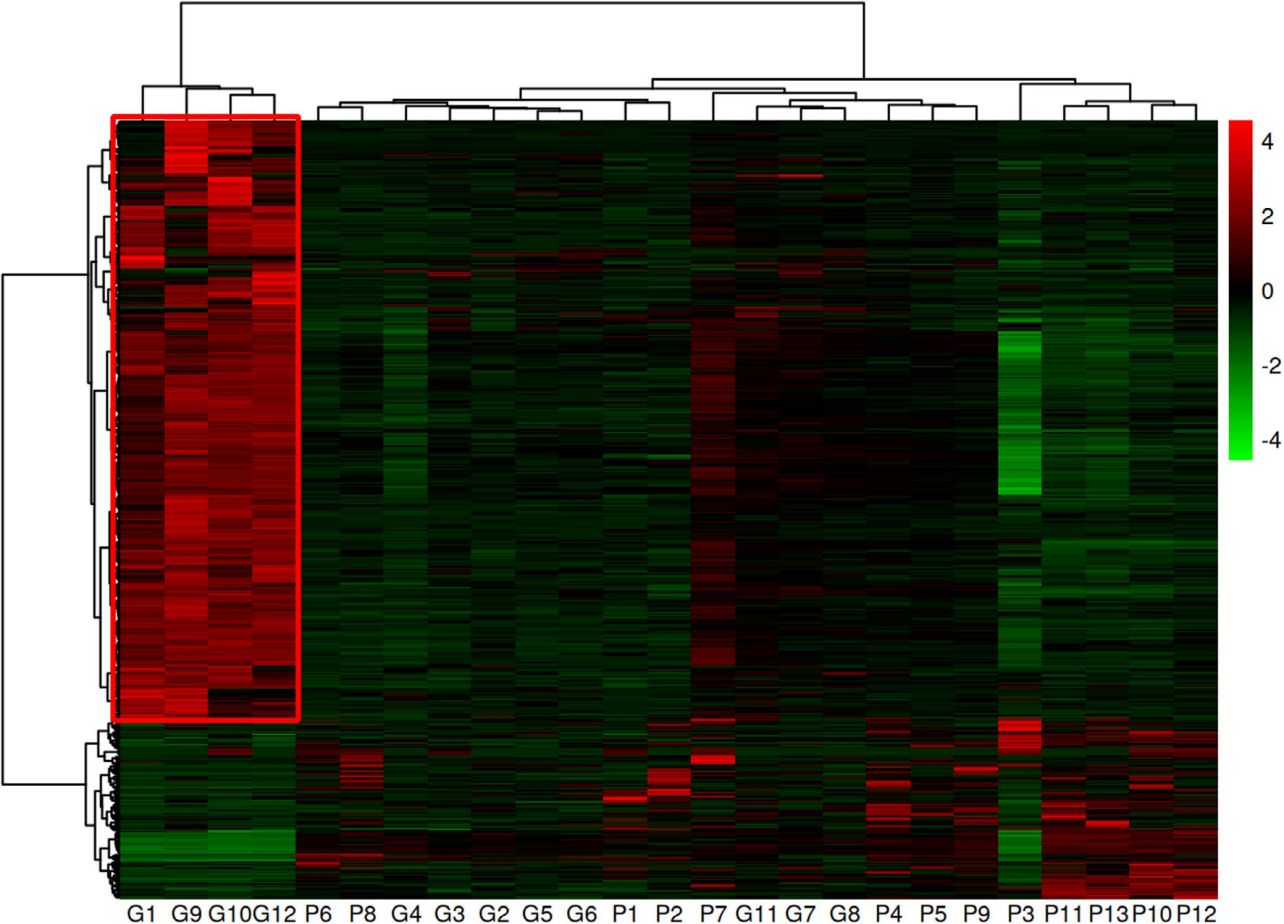
The heatmap show four patients within the good group demonstrate a significant level of clustering (highlighted by the red border).

**FIGURE 5:**
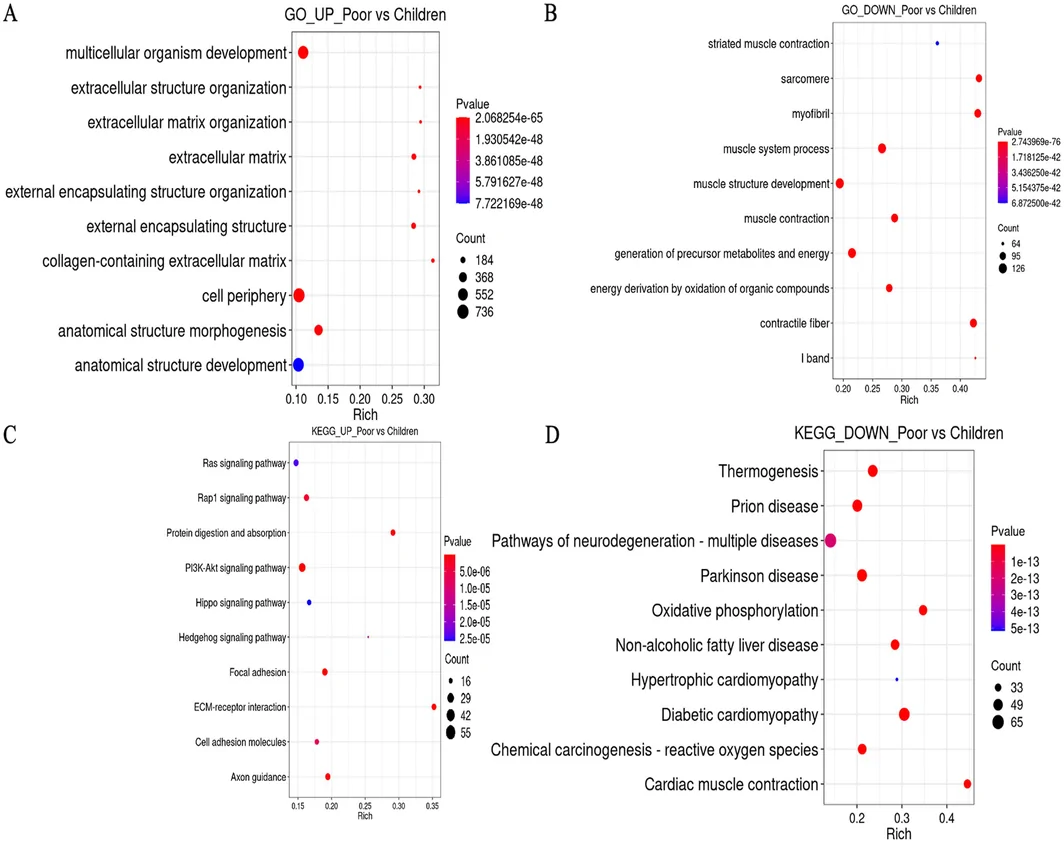
(A) Comparison of the paediatric subgroup (within Good group) vs. Poor group in GO and KEGG pathways. (A) Enrichment of upregulated genes in paediatric patients via GO analysis. (B) Enrichment of downregulated genes in paediatric patients via GO analysis. (C) KEGG pathway analysis of upregulated genes in the paediatric cohort. (D) KEGG pathway analysis of downregulated genes in the paediatric cohort.

Further exploration of the heatmap revealed that four patients within the Good group exhibited a high degree of clustering (highlighted by the red border in Figure 4), all of whom were children (< 18 years). We then performed a comparative transcriptomic analysis between these four children and the Poor group (*n* = 17, comprising 4 children and 13 adults). This analysis revealed 2392 DEGs, with 1425 upregulated and 967 downregulated. GO and KEGG pathway analyses indicated that the upregulated genes in children were strongly associated with mitochondrial electron transport chain activity and NADH dehydrogenase (Complex I) function (Figure 5A,C). Downregulated genes were mainly enriched in muscle‐related processes and energy metabolism according to GO analysis (Figure 5B), and were linked to thermogenesis and neurodegenerative diseases by KEGG analysis (Figure 5D). Interestingly, genes related to mitochondrial electron transport (e.g., NDUFS family) were among the differentially expressed genes in this small paediatric subset. Furthermore, PPI network analysis also highlighted the connectivity of these genes within the top‐ranked module, consistent with their coordinated expression pattern (Table 2). However, given the limited sample size, this finding should be interpreted with caution as a preliminary observation rather than a definitive mechanism.

### 3.4 Validation of ECM Remodelling by qPCR

**FIGURE 6:**
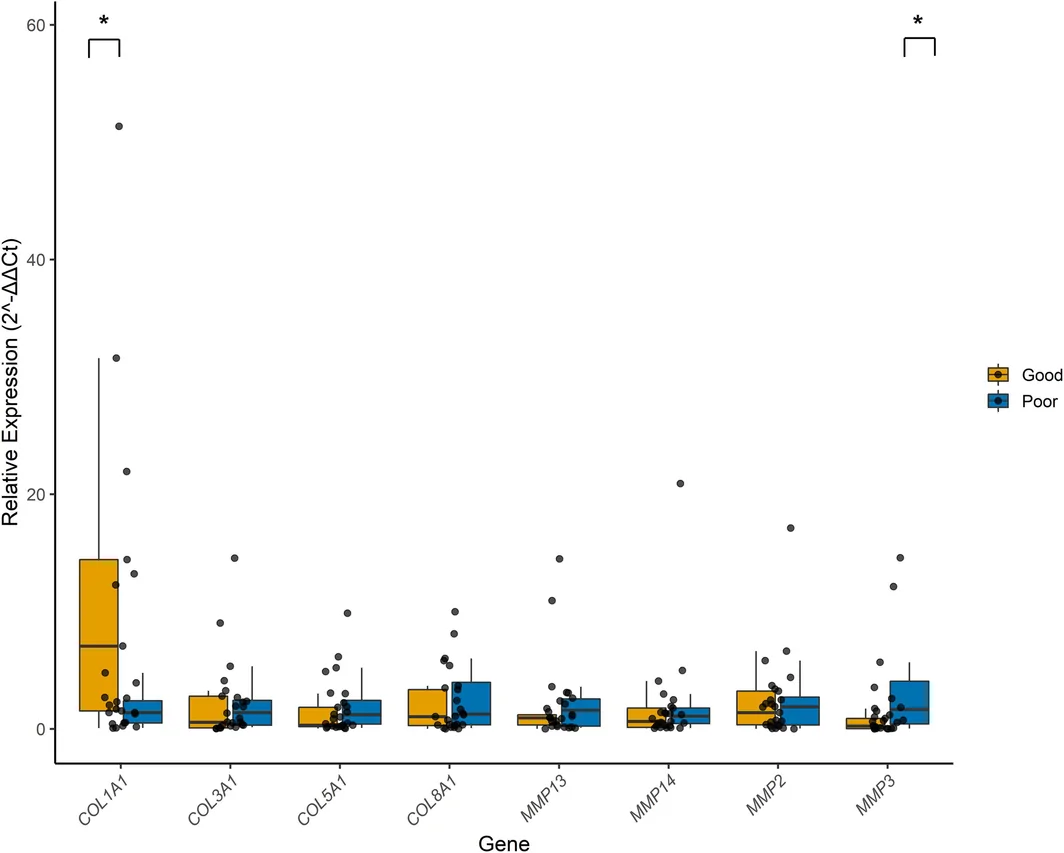
Relative expression of collagen/MMP genes showing notable differences between ‘Good’ and ‘Poor’ groups.

To validate the transcriptomic findings, we performed qPCR on genes involved in ECM (Figure 6). Consistent with the RNA‐seq data, COL1A1 expression was higher in the Good group compared to the Poor group (*p* = 0.044). Regarding matrix metalloproteinases, MMP3 expression also showed notable differences, being lower in the Good group (*p* = 0.041). These results support the notion that specific alterations in collagen expression (COL1A1) and MMP‐mediated remodelling (MMP3) may be involved in favourable postoperative angiogenesis.

## 4 Discussion

The therapeutic efficacy of indirect revascularization in MMD fundamentally partly relies on the ingrowth of new vessels from the temporal muscle into the cerebral cortex [19]. Accordingly, patients were stratified into Good and Poor groups based on the angiographic evidence of postoperative collateral formation. By comparing the transcriptomic profiles of the temporal muscle between these groups, we aimed to identify molecular correlates associated with successful or failed postoperative angiogenesis.

Transcriptomic analysis revealed differential expression of genes related to ECM remodelling. Based on an exploratory analysis, we observed that members of the collagen and MMP families may be potentially involved in this biological process. These findings suggest that the local microenvironmental characteristics of the temporal muscle represent one potential factor influencing the efficacy of indirect revascularization, although further studies with larger cohorts are required to validate this observation.

### 4.1 ECM And Collagen as Prominent Features of Postoperative Angiogenesis

ECM molecules, including collagen, laminin, and fibronectin, are not merely structural scaffolds; they actively promote endothelial cell survival, migration, and tube formation, while also recruiting vessel‐stabilizing pericytes and sequestering angiogenic factors to support vascular maturation [20, 21]. Previous studies have found that newly developed ECM tissues exhibit active angiogenesis between the temporal muscle and brain [22]. He et al. recently demonstrated, using spatial multi‐omics, that ECM remodelling and adhesion signalling (e.g., the FN1–ITGA5–FAK axis) are prominently involved in moyamoya disease vasculopathy [6]. Interestingly, our transcriptomic data identified a collagen‐rich ECM signature in the temporal muscle that was associated with increased postoperative angiogenesis, aligning with the concept of ECM activation in MMD vasculopathy.

Collagens constitute approximately 30% of mammalian protein mass and play pivotal roles in biomechanics and growth factor sequestration [23]. They enhance the strength and stability of the blood vessel wall while providing a matrix framework for new vessel sprouting [21]. Studies have demonstrated that collagen can induce angiogenesis during wound healing [24]. For example, the widely expressed COL1A1 provides a robust matrix for neovascularization [25, 26], whereas Type III collagen maintains vascular elasticity. In contrast, Type VIII collagen, localized at the basement membrane, specifically facilitates endothelial cell adhesion and migration during vascular repair [27, 28]. This coordinated upregulation underscores the necessity of a well‐organized ECM scaffold for postoperative neovascularization [23].

Our transcriptomic and qPCR validation supported that COL1A1 was notably upregulated in patients with favourable outcomes, suggesting that a collagen‐rich microenvironment may contribute to successful postoperative angiogenesis. Mechanistically, this upregulation in the temporal muscle may establish a more favourable matrix framework, potentially facilitating the postoperative ‘creeping’ of new vessels from the temporal muscle to the cerebral cortex.

From a clinical translation perspective, this finding implies that preoperative assessment of collagen (particularly COL1A1) expression levels in the temporal muscle could potentially serve as a biomarker for predicting the efficacy of indirect bypass surgery. Further, future studies could explore strategies such as local administration of collagen‐mimetic peptides or modulation of key enzymes in collagen metabolism to actively remodel the ECM of the transplanted muscle flap, thereby “empowering” the temporal muscle to enhance its pro‐angiogenic capacity. This provides a theoretical basis for developing novel therapeutic strategies to augment indirect revascularization.

### 4.2 MMPs And the Dynamic Balance of ECM Remodelling

ECM remodelling—the dynamic balance between synthesis and degradation—is essential for endothelial invasion. Notably, matrix metalloproteinases (MMPs), a family of calcium‐dependent zinc endopeptidases, drive this process by degrading collagen and exposing cryptic binding sites to promote angiogenesis [21]. Additionally, proteolytic cleavage of collagens releases bioactive fragments that further modulate angiogenic responses [29].

Our qPCR results revealed that MMP3 expression was significantly downregulated in the Good group. As a key member of the MMP family, MMP3 degrades various ECM components [30] and exhibits a dual role: while moderate expression supports normal ECM remodelling, its overexpression can trigger pathological proteolysis, foster a pro‐inflammatory microenvironment, and even induce endothelial cell apoptosis [31]. In this study, MMP3 expression was significantly higher in the Poor group compared to the Good group. This overexpression disrupts the balance of ECM remodelling, potentially reflecting pathological ECM degradation or an inhibitory state, thereby impeding effective collateral circulation formation. This difference suggests that the balance between specific MMPs (such as MMP3), rather than merely collagen abundance, may be a critical determinant of successful angiogenesis [32]. The differential expression of MMP3 suggests that assessing angiogenic potential requires evaluating not only collagen synthesis but also the balance among MMP family members.

### 4.3 Age‐Dependent Mechanisms: Mitochondria in Children

Consistent with previous reports, younger age correlated with better collateral formation postoperatively [10, 11]. Ageing impairs the adaptive response to hypoxia, thereby compromising postoperative angiogenesis; this phenomenon has also been verified in patients with MMD [33, 34]. In paediatric patients, the transcriptomic profile was dominated by genes related to mitochondrial electron transport (NADH dehydrogenase complex I). Reactive oxygen species (ROS) generated via mitochondrial respiration can promote angiogenesis [35, 36]. Our limited paediatric subset showed a trend towards enrichment in genes related to mitochondrial electron transport. While this aligns with the notion that children possess robust metabolic reserves, the small sample size precludes definitive conclusions regarding a causal relationship. This trend warrants further confirmation in future, larger‐scale studies.

## 5 Limitations

Limitations of the study include: (1) The Good group was considerably younger and had a lower prevalence of diabetes compared to the Poor group. Since ageing and diabetes are known to profoundly affect the ECM composition, inflammatory status, and angiogenic capacity of skeletal muscle, we cannot definitively exclude the possibility that these confounding factors, rather than collagen‐specific mechanisms, contributed to the observed transcriptomic differences. Future studies employing propensity score matching or multivariable regression models are required to isolate the specific effect of the collagen‐ECM axis. (2) The temporal muscle samples were collected at a single time point (during surgery) and cannot represent the dynamic changes occurring 1–6 months postoperatively during angiogenesis; (3) The small sample size and exploratory nature of the study may be susceptible to inter‐individual variability and batch effects; (4) The analysis focused on transcriptional changes, while post‐transcriptional, translational, and protein‐level alterations were not examined.

## 6 Conclusion

In conclusion, ECM components, especially collagen, appear to play an important role in temporal muscle angiogenesis. Further investigation into the underlying mechanisms by which collagen promotes this process is warranted. From a translational perspective, modulating the collagen‐ECM axis—such as through targeting MMP3 or supplementing specific collagen peptides—represents a potential avenue to improve surgical outcomes.

## Author Contributions

**Shun‐fei Xu:** software, formal analysis, data curation. **Ri‐sheng Liang:** supervision. **Xian‐kun Tu:** writing – review and editing, visualization, project administration, funding acquisition. **Jing‐yi Chen:** conceptualization, methodology, investigation, formal analysis, data curation, writing – original draft. **Zi‐qing Wang:** validation. **Wen‐xin Jin:** resources.

## Funding

The study was sponsored by the Fujian Provincial Health Technology Project (2021CXA015), the Excellent Young Scholars Cultivation Project of Fujian Medical University Union Hospital (2022XH040), the Fujian Provincial Natural Science Foundation (2023J01668), and the Fujian Research and Training Grants for Young and Middle‐aged Leaders in Healthcare.

## Conflicts of Interest

The authors declare no conflicts of interest.

## Data Availability

The transcriptome sequencing data generated in this study have been deposited in the NCBI SRA under BioProject accession PRJNA1111471 (https://www.ncbi.nlm.nih.gov/bioproject/PRJNA1111471). Additional data supporting the findings are available from the corresponding author upon reasonable request.
